# Supplementary material for: Evaluation of skin doses for cone-beam computed tomography in dentomaxillofacial imaging: A preclinical study
Source: PLoS One. 2021 Jul 12;16(7):e0254510. doi: 10.1371/journal.pone.0254510 (PMC8274873; doi:10.1371/journal.pone.0254510)
Supplement: S1 File — (DOCX) [file pone.0254510.s002.docx]

**Legend for our supporting information file**

**Column 1: Device**

1 = Dürr Vista Vox

2 = Morita 3D Accuitomo

3 = New Tom VGi evo

4 = Sirona Orthophos

5 = Planmeca Promax

6 = Carestream CS

**Column 2: Regions**

Region 1 Left parotid gland

Region 2 Right parotid gland

Region 3 Left lens

Region 4 Right lens

Region 5 Intracranial

Region 6 Thyroid gland

**Column 3: Measurements**

Measurements were repeated five times each (1 – 5).

**Columns 4 and 5:**

Skin organ doses for standard (column 4) and high dose settings (column 5).
