# Supplementary material for: Evaluation of skin doses for cone-beam computed tomography in dentomaxillofacial imaging: A preclinical study
Source: PLoS One. 2021 Jul 12;16(7):e0254510. doi: 10.1371/journal.pone.0254510 (PMC8274873; doi:10.1371/journal.pone.0254510)
Supplement: Attachment — Submitted filename: ResponseToReviewers.docx [file pone.0254510.s003.docx]

Dear Editor and Reviewers,

Thank you very much for your detailed review, which I really appreciate. Please find my comments below.

Best regards,

Carolin Reidelbach

**Journal Requirements:**

When submitting your revision, we need you to address these additional requirements.

1. Please ensure that your manuscript meets PLOS ONE's style requirements, including those for file naming.

- This was corrected accordingly.

2.  Thank you for stating in your Funding Statement:

"Wiebke Semper-Hogg is a consultant of Dürr Dental SE.
https://www.duerrdental.com/en/home/
The study was partly funded by Dürr Dental SE.
A grant number does not exist."

i) Please provide an amended statement that declares *all* the funding or sources of support (whether external or internal to your organization) received during this study, as detailed online in our guide for authors at [http://journals.plos.org/plosone/s/submit-now](about:blank).  Please also include the statement “There was no additional external funding received for this study.” in your updated Funding Statement.

- This was corrected accordingly.

ii) Please include your amended Funding Statement within your cover letter. We will change the online submission form on your behalf.

- This was corrected accordingly.

3.Thank you for stating the following in the Competing Interests section:

"I have read the journal's policy and the authors of this manuscript have the following competing interests:
Wiebke Semper-Hogg is a consultant of Dürr Dental SE.".

 i) Please confirm that this does not alter your adherence to all PLOS ONE policies on sharing data and materials, by including the following statement: "This does not alter our adherence to  PLOS ONE policies on sharing data and materials.” (as detailed online in our guide for authors [http://journals.plos.org/plosone/s/competing-interests](about:blank)).  If there are restrictions on sharing of data and/or materials, please state these. Please note that we cannot proceed with consideration of your article until this information has been declared.

- This was corrected accordingly.

ii) Please include your updated Competing Interests statement in your cover letter; we will change the online submission form on your behalf.

- This was corrected accordingly.

**Reviewer #1: Comments to the authors**

Aim of the study:
The last paragraph in the introduction is ‘’the purpose of this study was to do further research on this topic, as side differences seem to be expectable owing to the applicational and structural differences between the devices on the market’’. Then, the authors wrote in the second paragraph in the discussion ‘’ The aim of this study was to examine side differences in distribution of dose’.
The aim was not precisely described in this manuscript. It is general and the authors have to be more specific.

- The aim of the study was described more precisely in the introduction and discussion section.

methodology
The authors evaluated the ‘’side differences’’, however, they did not clarify to the readers what is/are the side difference/s, moreover, in the methodology they did not describe how they measured the side differences. The authors should describe in details the side differences and the method which they applied to measure those differences.

- This was corrected accordingly in the methods section.

results
The authors did describe the distribution of dose in the result section. They should point to the figure that describes the distribution of dose.

- This was corrected accordingly.

**Reviewer #2: Dear authors**

In the Discussion section : please answer the questions and do the needed corrections listed below:

1- you have to start the section by the importance and the clinical consideration of conducting your study following by mentioning the aim of the study.

- The beginning of the discussion section was corrected accordingly.

2- you have to explain the causes of using each material and method items like this type of phantom, these types of CBCT devices, 3D dosimeter and organs (parotid, eye lens, thyroid and neurocranium).

- The causes of using each material were amended accordingly in the methods section and partially in the discussion section.

3- what is your explanation about achieving the highest mean organ dose by Morita 3D Accuitomo 170?

- An explanation was amended accordingly.

4- side differences in distribution of dose for the parotid and eye lens were confirmed by previous research please mention them and put a reference on your explanation of that difference.

- This was corrected accordingly.

5- please clarify the explanation of side difference in a full rotation mode ( New Tom VGi evo) and mention the other devices with similar findings not only example.

- This was corrected accordingly.

6- of the eight lines of explanation on the first paragraph at the second page of the discussion section (page 9), please add a references for that explanation and rewrite it again to be more clarify.

- This was corrected accordingly.

7- At the end of the above paragraph you mention that " in literature only few studies...) please add more references and give the clinical consideration about this type of studies especially in dentistry.

- This was corrected accordingly.

8- fifth line in the second paragraph at the second page of the discussion section (page 9) to the end of the paragraph you write a lot of information without references please add references and relate with your findings.

- This was corrected accordingly.

9- the six lines of the third paragraph in the discussion section (page 9) have no references please add references.

- This was corrected accordingly.

10- line No seven in the third paragraph of the discussion section (page 9) refer to the cause of high dose to the parotid glans this is repeated information as it mentioned in the previous paragraph therefore, please rewrite it in a concise manner.

- This was corrected accordingly.

11- in the fourth paragraph of the discussion section (page 9) you mention only one old reference [10] please clarify how the finding in this reference relate to your work

- This was corrected accordingly.

12 some spelling errors at the sixth line in the second paragraph of the discussion please correct (und) between parotid and lens. another error in the 7th line of the second paragraph in page 9 (parotis)

- This was corrected accordingly.

13 please rewrite the discussion section because there is lack of flow and organization in the text .

- This was corrected accordingly.

14 lastly please use constrained statements to explain the aim of study, limitation and clinical consideration.

- This was corrected accordingly.

**Reviewer #3:**

Q1what's you think this work add to the subject area compared with other published work?.

- A statement on this question was amended accordingly in the discussion section.

Q2 Some CBCT in your research don't have high dose setting ?how manage this missing data ? for comparison purposes?.

- A statement on this question was amended accordingly in the discussion section.

Q3 In the abstract ,conclusion section the last row (further studies should be performed to confirm these result ) this is your conclusion or suggestion?

- As a final statement in the discussion section, this is our conclusion.

Q4 In the introduction section(the main drawback of cross sectional imaging is the higher radiation dose compered to radiography ) which type of radiography?.

- We meant conventional radiography - this was corrected accordingly.

Q5 In method , dose measurement section can you descried in details standard dose setting and high dose setting?.

- Standard and high dose settings with detailed parameters were listed in table 1.
